# Characterization and Proteomic Analysis of Plasma EVs Recovered from Healthy and Diseased Dogs with Canine Leishmaniosis

**DOI:** 10.3390/ijms24065490

**Published:** 2023-03-13

**Authors:** Sofia Esteves, Clara Lima, Inês Costa, Hugo Osório, Carmen Fernandez-Becerra, Nuno Santarém, Anabela Cordeiro-da-Silva

**Affiliations:** 1 Instituto de Investigação e Inovação em Saúde, Universidade do Porto, 4200-135 Porto, Portugal; 2 Laboratório de Microbiologia, Departamento de Ciências Biológicas, Faculdade de Farmácia da Universidade do Porto, 4050-313 Porto, Portugal; 3 Ipatimup—Institute of Molecular Pathology and Immunology of the University of Porto, University of Porto, 4200-135 Porto, Portugal; 4 Department of Pathology, Faculty of Medicine, University of Porto, 4200-319 Porto, Portugal; 5 ISGlobal, Barcelona Institute for Global Health, Hospital Clínic-Universitat de Barcelona, Carrer Rosselló 149-153, CEK Building, 08036 Barcelona, Spain; 6 IGTP Institut d’Investigació Germans Trias i Pujol, Ctra. de Can Ruti. Camí de les Escoles, S/n, Badalona, 08916 Barcelona, Spain; 7 CIBERINFEC, ISCIII-CIBER de Enfermedades Infecciosas, Instituto de Salud Carlos III, 28029 Madrid, Spain

**Keywords:** canine leishmaniosis, *Leishmania infantum*, extracellular vesicles, proteomics, exosomes, leishmaniasis, *Leishmania*, dogs, parasites

## Abstract

Dogs are highly valued companions and work animals that are susceptible to many life-threatening conditions such as canine leishmaniosis (CanL). Plasma-derived extracellular vesicles (EVs), exploited extensively in biomarker discovery, constitute a mostly untapped resource in veterinary sciences. Thus, the definition of proteins associated with plasma EVs recovered from healthy and diseased dogs with a relevant pathogen would be important for biomarker development. For this, we recovered, using size-exclusion chromatography (SEC), EVs from 19 healthy and 20 CanL dogs’ plasma and performed proteomic analysis by LC-MS/MS to define their core proteomic composition and search for CanL-associated alterations. EVs-specific markers were identified in all preparations and also non-EVs proteins. Some EVs markers such as CD82 were specific to the healthy animals, while others, such as the Integrin beta 3 were identified in most samples. The EVs-enriched preparations allowed the identification of 529 canine proteins that were identified in both groups, while 465 and 154 were only identified in healthy or CanL samples, respectively. A GO enrichment analysis revealed few CanL-specific terms. *Leishmania* spp. protein identifications were also found, although with only one unique peptide. Ultimately, CanL-associated proteins of interest were identified and a core proteome was revealed that will be available for intra- and inter-species comparisons.

## 1. Introduction

Several studies have shown that extracellular vesicles (EVs) contain proteins and nucleic acids, associated with several disorders, including infectious diseases [1]. EVs carry cell-specific molecular information protected by a double membrane structure. Compared to other conventional soluble biomarkers detected in biological samples, the EVs provide the promise of specificity and sensitivity comparable, or even higher, due to their excellent biological stability. It should also be stated that EVs are a privileged source of membrane-associated proteins, that are not found free in biological fluids. Moreover, EVs can be recovered from easily obtained bio fluids such as blood, saliva, tears, or urine making them invaluable in clinical applications. Thus, EVs have been the subject of many scientific efforts to provide the next generation of clinically relevant biomarkers [2]. Still, few studies in veterinarian sciences report EVs characterization and evaluation as potential biomarkers. Particularly, studies using EVs recovered from dogs are scarce. Some reports studied EVs produced in vitro, such as the proteomic characterization of EVs derived from canine oviductal cells [3]. In addition, a proteomic profile of exosomes from healthy osteoblasts and osteosarcoma cell lines was described as well as their impact on healthy T cells [4]. Tissue explants were also taken from dogs with osteosarcoma and EVs were recovered in vitro after 24 h. A posterior proteomic analysis followed and two molecular targets for this disease were identified—PSMD14/Rpn11 [5]. In the context of biological fluids, plasma-derived exosomes from osteosarcoma-bearing dogs, healthy dogs, and dogs with traumatic fractures were characterized, in conjunction with patients with osteosarcoma prior and 2 weeks after amputation. This characterization allowed the identification of a group of proteins capable of distinguishing healthy dogs with natural fractures from dogs with osteosarcoma [6]. EVs recovered from obese female dogs’ serum were recovered and their miRNAs profile was evaluated [7]. The available information suggests that, much like EVs from humans, EVs from dogs might be used to address the clinical condition of the animals and unravel disease-specific biomarkers. In this context, vector-borne canine leishmaniosis (CanL), caused by *Leishmania infantum* protozoan parasites, is a major concern for the veterinary community, having high mortality and morbidity rates [8]. In endemic areas, domestic dogs are considered the main reservoir of *L. infantum*, as they allow parasite life cycle perpetuation. The relation between the incidence of CanL and human zoonotic leishmaniasis has been described, so CanL is not only considered a veterinary problem but also a public health issue [9]. Therefore, considering the zoonotic potential of *Leishmania* infection, disease management in dogs is essential from the One Health perspective. The available tools, in conjunction with clinical evaluation, are adequate to detect diseased dogs but present limitations when trying to diagnose infection in asymptomatic dogs [10]. Thus, the real prevalence and overall risk of infection are believed to be underestimated [11]. The current diagnosis options are suboptimal, so there is a need for alternative tools that are able to address complex situations such as asymptomatic disease, co-infection, and treatment assessment [12]. To achieve this, we must overcome the limitations concerning the availability of biological samples from infected tissues and mixed serology results that are highly dependent on the antigens used [13]. Molecular approaches using host/parasite-specific molecules would be the ideal scenario. In this context, EVs are a resource with great potential [2], as these small lipidic vesicles of cellular origin are present in all biological fluids and have great stability. In fact, the potential of EVs as a source of disease-relevant biomarkers has been proposed for other parasitic pathogens [14,15,16,17,18]. EVs from *Leishmania* have been previously characterized [19,20] and parasite-associated proteins were also identified in infected macrophages [21,22,23]. Moreover, circulating parasite material was found in the urine of visceral leishmaniasis patients [24] as well as in immunocomplexes [25]. Specifically, in the context of CanL, EVs were recovered from the canine serum of healthy and diseased dogs and the exosomal miRNA 122 expression was evaluated. The levels of miRNA 122, a marker of liver impairment, decreased in diseased dogs [26]. Nonetheless, the protein cargo of plasma-derived EVs was not yet characterized in healthy or CanL dogs. This study represents the first systematic report of characterization and proteomic analysis of circulating EVs recovered from canine plasma with the goal of defining the core proteome of these EVs and evaluating plasma EVs as a potential source of biomarkers relevant for CanL. This study will pave the way to the field of EVs in these companion animals. It will enable future inter-species comparisons and also help define EVs as a source of potentially relevant biomarkers for CanL and other relevant clinical conditions.

## 2. Results

### 2.1. Generic Sample Characterization

Thirty-nine dogs were included in this study. These were characterized clinically and grouped into CanL and/or Healthy groups (Table 1; Figure S1). The Healthy group was composed of dogs presenting unremarkable physical examination, negative serological detection of anti-*Leishmania* antibodies performed by a rapid chromatographic test validated for anti-*Leishmania* antibodies detection and an in-house ELISA for SPLA and rK39, besides negative blood-PCR for *L. infantum*. In this group, 14 dogs resided in CanL non-endemic regions (11 dogs from Ireland, 3 dogs from Azores) and 8 dogs were from Portugal. The samples included in the CanL group belonged to dogs living in CanL-endemic regions of Portugal that were found polysymptomatic for CanL (Figure S1). The diagnosis was confirmed either through commercially available serological tests validated for CanL diagnosis, together with in-house ELISA for SPLA and rK39, or parasite isolation from blood, lymph nodes, or bone marrow. A complete blood cell count (CBC) was performed in 18 CanL dogs (Figure S1).

### 2.2. EVs recovery and Characterization 

**Figure 1:**
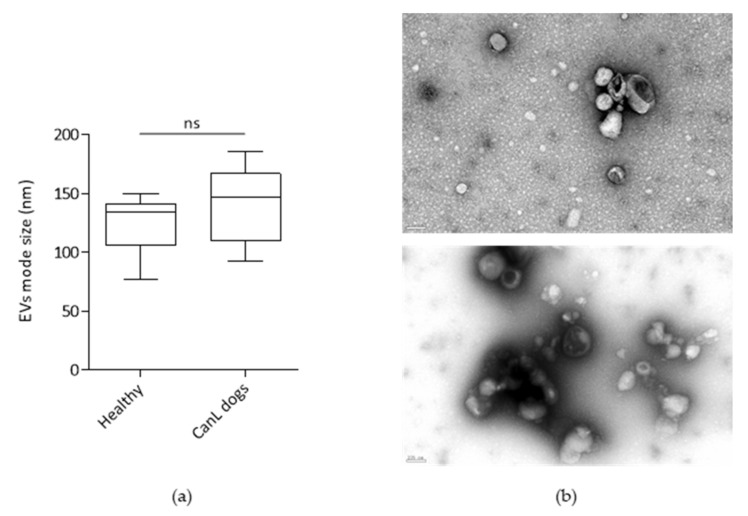
(**a**) Characterization of EVs mode size by NTA of the Healthy group (n = 8) and CanL group (n = 12). Statistical significance was determined using Mann–Whitney test. ns—non-significant; (**b**) representative images of plasma-derived EVs recovered by SEC from a CanL dog (**upper** image) and healthy dog (**lower** image) obtained with negative staining transmission electron microscopy, using Jeol JEM 1400 with STEM detector and EDS system. The bar in the lower left corner of the images represents 100 nm.

After sample selection, plasma was processed by size exclusion chromatography (SEC) using 1 mL sepharose columns. The obtained fractions were characterized by protein quantification and bead-based assay, using CD9, CD71 and CD5L as EV-markers. The CD9 is a classical tetraspanin, used in several studies to characterize EVs including in the context of exosomes of dogs serum characterization [26], CD5L is a marker previously shown to be present in human plasma-derived EVs and in the context of isolation of EVs from a patient with Chagas disease [17,27] and CD71 is an exosomal marker previously used in the context of characterization of plasma-derived EVs in *Plasmodium vivax* infected patients [28,29]. The fractions with the highest median fluorescent intensity (mfi) in the bead-based assay, enriched in the EV markers, were pooled (Figure S3). The pooled fractions were further characterized by NTA for size determination, by TEM and mass spectrometry for protein identification of *Canis lupus familiaris* and *L. infantum* proteins (Figure 1).

### 2.3. Qualitative Evaluation of Canine Proteins 

**Figure 2:**
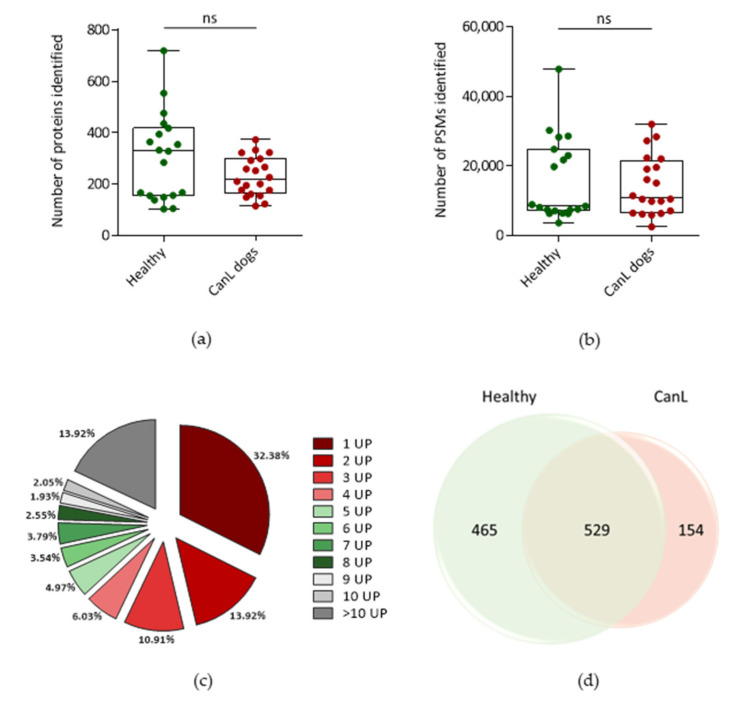
Number of (**a**) canine proteins and (**b**) spectrums—PSMs identified for each EVs sample in the healthy animals and in CanL animals. Statistical significance was accessed using Mann-Whitney test. ns—non-significant; (**c**) number of unique peptides associated with each protein identification. (**d**) Venn diagram of the number of proteins identified in the merge of CanL group versus Healthy group. Only proteins with 2 or more unique peptides and a maximum false discovery rate (FDR) of 1% for peptides were included.

The average number of proteins identified in each sample was 305 and 230 for the Healthy group and CanL group, respectively, while the average number of Peptide-Spectrum Matches (PSMs) was 15,859 and 14,390 (Figure 2a,b). 

When comparing the CanL and the Healthy groups, no significant differences in the number of proteins or PSMs were observed (Figure 2a, Table S1). Identifications with 1 unique peptide (UP) were not considered in the analysis, although represented most identifications, 32.38% (Figure 2c). More than 53.6% of the proteins were identified with 3 or more UP. A comparative proteomic analysis was performed using the merged data from each group (merged data from the 20 CanL EVs and the merged data from the 19 healthy dogs EVs) (Figure 2d, Table S2). 

Considering the total identifications from the merged CanL compared to the merged Healthy groups, a total of 1148 canine proteins were identified with high confidence. A total of 529 canine proteins were common to both conditions, corresponding to 46.1% of the total proteome identified. Moreover, 154 canine proteins were only identified in the CanL EVs, and 465 proteins were only identified in the Healthy dogs’ EVs (Table S2).

**Figure 3:**
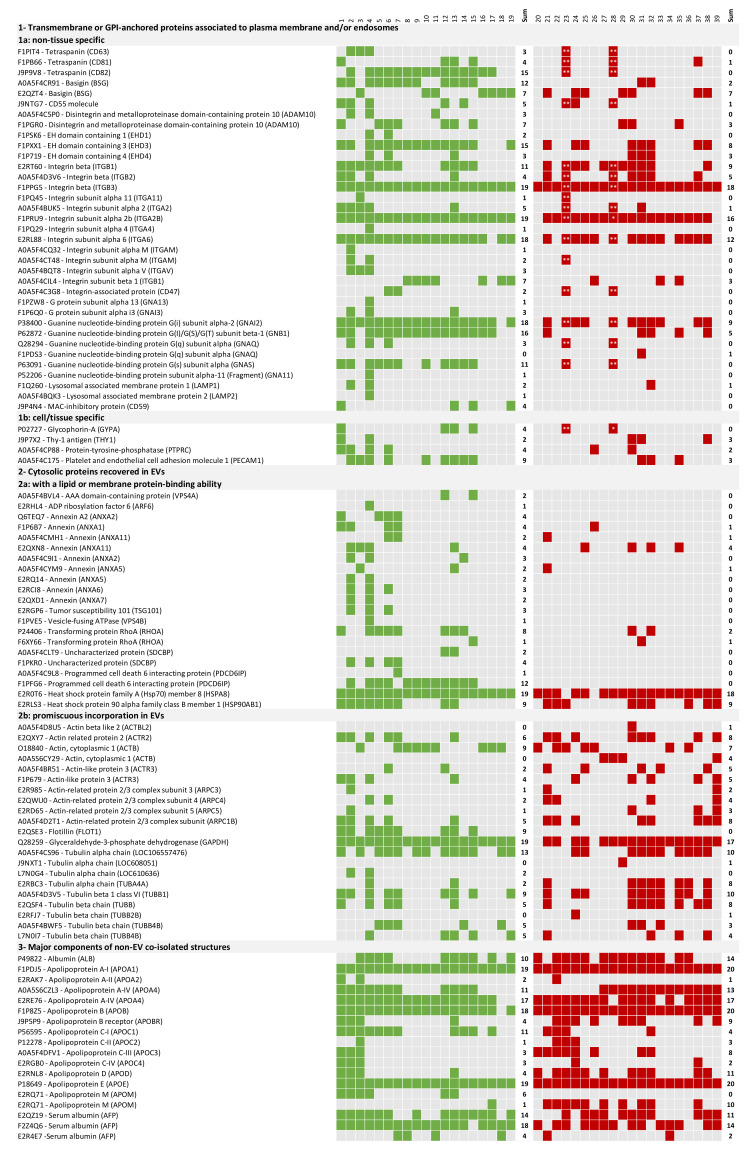
Protein content-EV characterization based on the MISEV2018 guidelines. Each line represents a protein, while the columns labelled with a number represent the individual samples. The presence of green (Healthy group) or red (CanL group) colors associated to individual proteins depicted in lines represents the detection of that protein in the sample with at least 2 unique peptides. For samples 23 and 28, proteins with one unique peptide (*) and spectrums identified (**) were also considered.

To confirm that the fractions sent to proteomics were enriched in EVs, the proteomic characterization was performed according to MISEV2018 guidelines [30] (Figure 3). In all preparations, at least one protein of categories 1, 2 and 3 was detected. 

Concerning the recommended EVs markers, among the transmembrane or GPI-anchored proteins associated to plasma membrane and/or endosomes, the Integrin alpha (ITGA) and beta (ITGB) were the most consistently identified in the individual samples. In fact, ITGB3 was the most consistently detected. Most markers were only sporadically detected, while others such as CD82 were mostly detected in the Healthy cohort. Among the cytosolic proteins recovered in EVs, the Heat Shock Protein family A (HSP70) member 8 (HSPA8) was the most consistently detected. Among proteins with promiscuous incorporation in EVs, Gliceralehyde-3-phosphate dehydrogenase (GAPDH) was the most consistently detected. Several non-EV co-isolated structures were also detected, such as the apolipoprotein E that was detected in all samples (Figure 3 and Figure S4). Among the other proteins recommended by the MISEV2018 guidelines, CD5L was identified in all preparations. 

**Figure 4:**
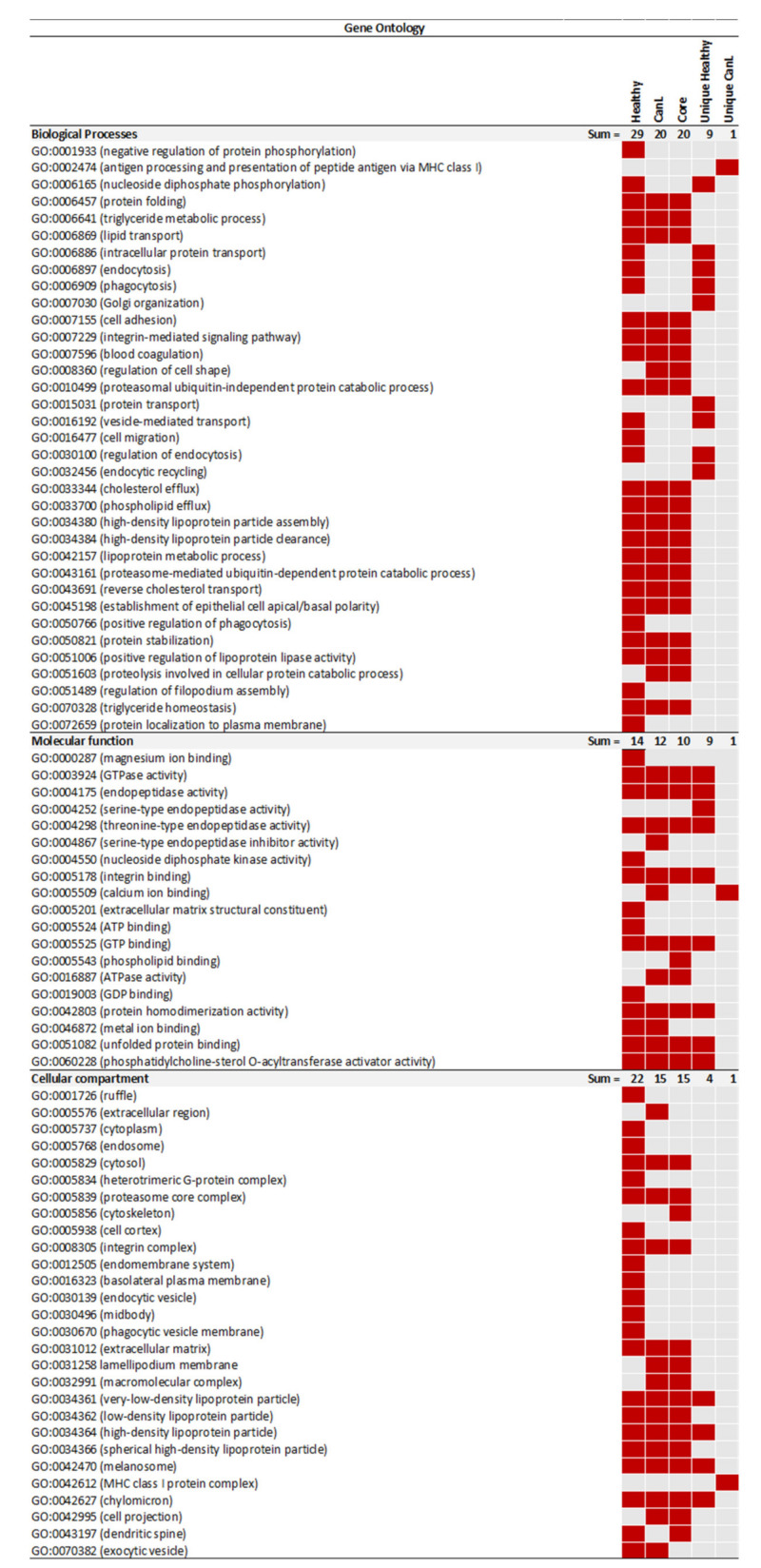
Gene ontology (GO) enrichment analysis of the proteins in the Healthy dogs EVs, CanL EVs, in the core proteome, in the proteins unique to Healthy EVs and proteins unique to CanL EVs. GO enrichment analysis shows terms significantly enriched for biological processes, molecular function and cellular compartment.

To evaluate the biological impact of CanL in the EVs containing fractions, a GO enrichment analysis using the Database for Annotation, Visualization, and Integrated Discovery (David 2021) was performed (Figure 4, Table S3). This analysis was performed at two levels; first, a simple comparison of the Healthy and CanL group, then, a subsequent analysis based on common (core proteome) and group-specific protein identifications. The proteins identified in the EVs containing fractions from healthy dogs were associated to 29 significantly enriched biological processes, while for the CanL group there were 20. From the biological processes significantly enriched in CanL, 18 were present also in the Healthy group. Considering the proteins identified in the core proteome, 20 biological processes were found significantly enriched, matching perfectly the biological processes enriched in CanL samples. Considering the analysis performed using the proteins that were uniquely identified in each group, nine GO terms were found to be significantly enriched in the Healthy group, and a single GO term associated to CanL EVs, the antigen processing and presentation of peptide antigen via MHC class I (GO:00002474). Among the Healthy-specific GO terms, three were not significantly enriched when considering the complete Healthy group data set: endocytic recycling (GO:0032456), Golgi organization (GO:0007030) and protein transport (GO:0015031). When molecular function was considered, 14 GO terms were found significantly over-represented in the complete Healthy data set, and 12 for the complete CanL data set. Among the 12 GO terms associated to molecular function that were enriched in the complete CanL data set, three were not found significantly enriched in the complete Healthy data set: serine-type endopeptidase inhibitor activity (GO:0004867), calcium ion binding (GO:0005509) and ATPase activity (GO:0016887). The latter, GO:0016887, was also significantly enriched in the core proteome. Calcium ion binding (GO:0005509) was also the only term significantly over-represented in the CanL unique proteins. Considering the Heathy unique proteins, nine GO terms associated to molecular functions were found to be enriched. Among these, eight were also found in the core proteome. Only the GO term associated with serine-type endopeptidase activity (GO:0004252) was not significantly enriched in the core proteome. Considering the GO analysis for cellular compartment, 22 terms were found enriched in the Healthy group, and 15 on the CanL. Among these 15, only 1, extracellular region (GO:0005576), was not present in the core proteome. Among the GO terms significantly enriched in the group-specific proteins, four were associated to the unique Healthy proteins. These terms were also enriched in the core proteome. For the unique CanL proteins only one GO term was enriched, MHC class I protein complex (GO:0042612).

**Figure 5:**
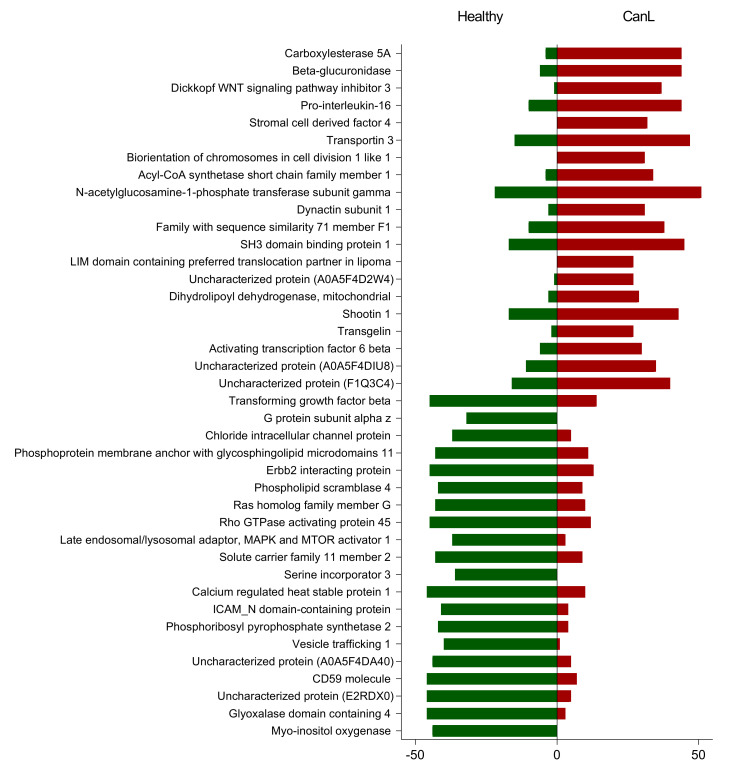
List of the 40 proteins that were mostly detected in either the injections from Healthy dogs (green columns) or CanL dogs (red columns). A total of 100 injections was considered (48 injections for 19 healthy samples and 52 for 20 CanL samples). For each protein identified, we included description and accession number from UniProt for uncharacterized proteins.

To evaluate the protein identifications in individual runs, the entirety of the 100 injections (48 injections for 19 healthy samples and 52 for 20 CanL samples) in the merged file were used to evaluate the capacity to detect the individual proteins (Table S4). The protein that was most consistently differentially detected in the injections was Myo-inositol oxygenase (E2QTD8), detected in 44 injections associated to healthy samples and none of the CanL (Figure 5). On the contrary, Carboxylesterase 5 A (A0A5F4BXK6) was detected in 40 out of the 52 injections of the CanL and only 4 of the Healthy injections. Moreover, stromal cell derived factor 4 (SDF4) and biorientation of chromosomes in cell division 1 (BOD1L1) were only detected in CanL injections, with 32 and 31 spectra identifications, respectively.

### 2.4. Quantitative Evaluation of Canine Proteins

**Figure 6:**
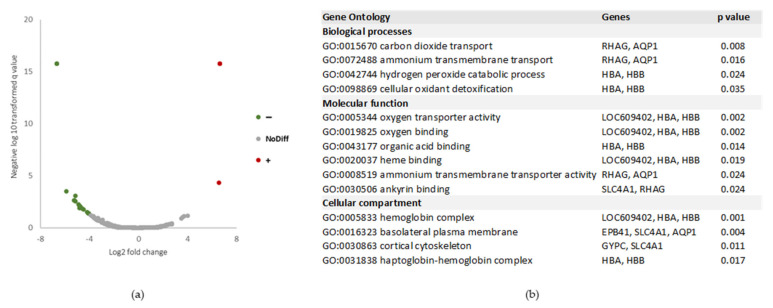
(**a**) Volcano plot representation of the abundance ratio of proteins present in CanL group versus Healthy group, represented in log_2_ fold change values. The differentially abundant proteins (*p* < 0.05) are represented by coloured dots. NoDiff—proteins with no altered abundance; “+”—proteins significantly more abundant in CanL group; “−“—proteins significantly less abundant in CanL group. (**b**) GO enrichment analysis shows terms for biological processes, molecular function and cellular composition significantly overrepresented in the proteins significantly less abundant in CanL group.

A quantitative analysis was also performed to compare the protein abundance between the 2 conditions, using all 100 individual injections. This analysis resulted in an abundance ratio (CanL/Healthy) and a *p* value associated with each protein. A distinct protein profile was revealed between the 2 groups and proteins with significantly altered abundance were identified: 5 proteins were significantly more abundant in CanL group and 24 more abundant in the Healthy group (Figure 6 and Table S5). The GO terms associated with the proteins more abundant in the Healthy group are mostly associated with red blood cells and oxygen/carbon dioxide transport. 

### 2.5. Canine Derived Biomarkers of CanL

**Figure 7:**
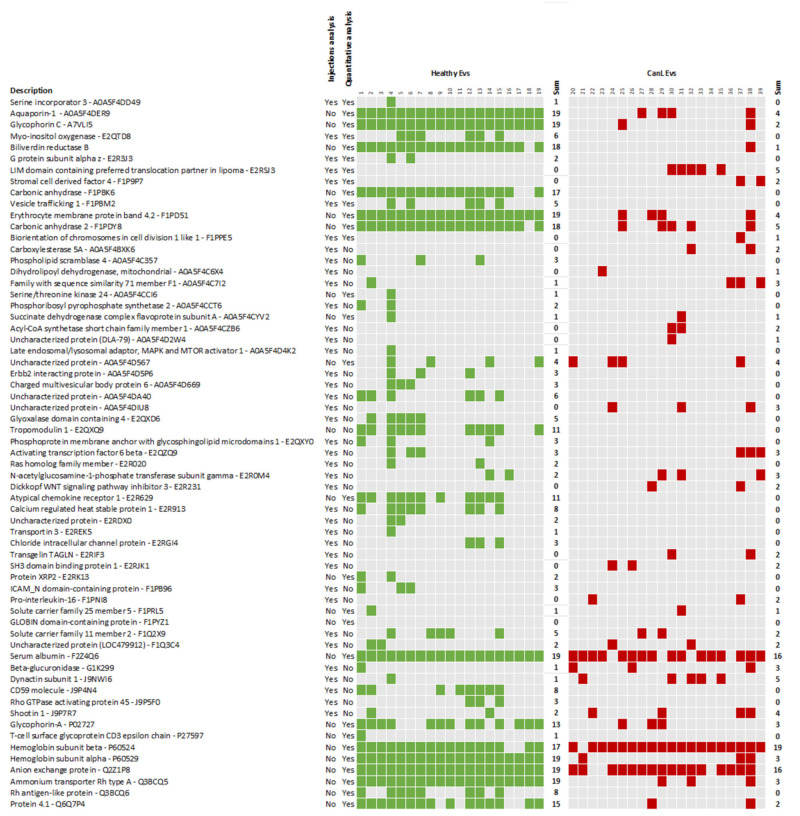
Detection of the most promising proteins present in individual samples. The selected proteins include all differentially abundant proteins identified in the quantitative analysis and the 40 proteins that were mostly detected in either the 48 injections from 19 Healthy dogs or the 52 injections from 20 CanL dogs. The second and third columns indicate if the proteins were identified by injection analysis, quantitative analysis or by both approaches. The presence of green (Healthy group) or red (CanL group) colours associated to individual proteins represents the detection of that protein in the sample with at least 1 unique peptide.

A retrospective analysis was then performed to evaluate the capacity to detect the most promising proteins from Figure 5 and Table S5 in the individual animals using as criteria for detection the presence of at least 1 UP (Figure 7). Among all the proteins the carbonic anhydrase (F1PBK6) was the best performer, being identified in 17/19 healthy animals and none of the CanL animals. Several other proteins of interest can be associated to the Healthy group, including biliverdin reductase B, present in 18/19 healthy samples and only 1 CanL sample, followed by glycophorin C present in all healthy samples although also present in 2 CanL samples. These three proteins cover all Healthy samples and only one CanL sample was associated with more than one of these proteins. 

### 2.6. Qualitative Evaluation of Leishmania Proteins

**Figure 8:**
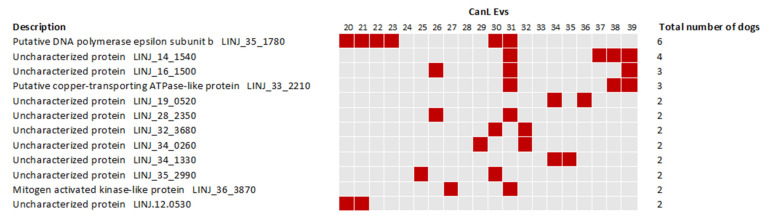
*Leishmania infantum* proteins identified in CanL individual samples. The presence of a red colour associated to individual proteins represents the detection of that protein in the sample with 1 unique peptide.

**Figure 9:**
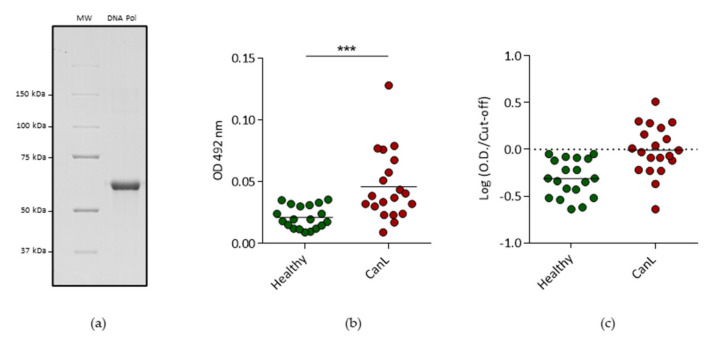
(**a**) SDS-PAGE analysis of the recombinant putative DNA Polymerase epsilon subunit b after expression at 37 °C, inclusion bodies isolation and solubilization and protein extraction. The band represented in the SDS-PAGE corresponds to 2.5 µg of the recombinant protein. MW—molecular weight; DNA Pol—putative DNA Polymerase epsilon subunit b; (**b**) Reactivity of sera from healthy and CanL dogs to putative DNA Polymerase epsilon subunit b determined by ELISA. Coating was done with 5 µg/mL of recombinant protein. Results are represented as the optical density at 492 nm. Data was generated from the average of two independent assays. Statistical significance was determined using the Mann–Whitney test. ***—*p* < 0.001 (**c**) Reactivity of sera from healthy and CanL dogs to putative DNA Polymerase epsilon subunit b determined by ELISA. Results are represented as the logarithm of the optical density (OD) at 492 nm normalized by the populational cut-off value (mean of the OD of the healthy samples + 2 standard deviations) for each antigen. The dashed line represents the cut-off. Data was generated from the average of two independent assays.

We also investigated the presence of *L. infantum* associated proteins in the samples, as the identification of parasite proteins would be relevant for diagnosis. No protein identification was supported by more than 1 UP. All identifications associated to the Healthy samples were excluded and only identifications unique to the CanL group were considered (Table S6). To evaluate if there was an enrichment of *Leishmania*-specific identifications, two other pathogens databases were used as controls in the proteomic data analysis. Considering that no identification with more than 1 UP was obtained, we evaluated the total number of spectra associated with the peptides detected in the Healthy and CanL and compared it with two other unrelated pathogens, *Neospora caninum,* a possible dog pathogen endemic to Portugal and *Plasmodium falciparum,* a non-dog pathogen non-endemic to Portugal (Figure S6). Only for *L. infantum*, the number of PSM/unique peptides was at least two times higher for the CanL group when compared to the Healthy group. For the two other pathogens databases, the identifications were similar in both groups. Considering the above mentioned constraints, 88 proteins were identified in the 20 CanL samples. Twelve proteins were identified in more than one dog (Figure 8 and Table S7). Putative DNA Polymerase epsilon subunit b (LINJ_35_1780) was detected in six dogs. Three other proteins were detected in more than two animals, the uncharacterized proteins (LINJ_14_1540) and (LINJ_16_1500) were detected in 4 and 3 animals respectively while the Putative copper-transporting ATPase-like protein (LINJ_33_2210) was also detected in three animals. The detected peptides associated with the identification of these 12 proteins were subjected to homology evaluation to further support the confidence of their association with *Leishmania* spp. (Table S8). Finally, the DNA Polymerase epsilon subunit b was also produced as a recombinant protein (Figure 9) and the presence of antibodies that recognize this protein was confirmed in the CanL group by a significant seroreactivity. Furthermore, a cut-off was determined and 9 samples were considered above the cut-off, of which 5 identified putative DNA Polymerase epsilon subunit in CanL EVs through proteomic analysis.

## 3. Discussion

The present study described the recovery, characterization and proteomic analysis of plasma-derived EVs purified by SEC from healthy and canine leishmaniosis dogs. No differences were seen in the mode size of EVs when comparing the two groups. However, different EVs sizes were already reported in the context of inflammation, tissue damage, cellular stress and infection [31,32]. This suggests that CanL does not cause a significant shift in EVs size as was observed for viral infections caused by HTLV-1. Considering the overall protein identifications, no differences were observed comparing the total number of protein identifications and PSM, suggesting that the diseased condition did not impact the overall capacity to detect proteins in the fractions of interest. Interestingly, the healthy group presented an apparent clustering into lower protein identifications and higher protein identifications. This apparent clustering was not attributable to any evaluated characteristic of the samples. The approach selected enabled a reproducible and consistent recovery of EVs-enriched preparations. These EVs-enriched populations are expected to constitute a mixture of EVs from different origins such as exosomes and microvesicles. Still, these preparations contained several abundant proteins in plasma, such as albumin and fibrinogen. This was a limitation of the approach. In fact, using the list from Menezes-Neto [27] about 25% of the detected spectra are associated to common plasma contaminants or non-EVs co-isolated proteins such as apolipoproteins. Moreover, no consistent group-specific enrichment in plasma-soluble proteins was observed. The only exception was albumin, which is in agreement with the hypoalbuminemia often observed in the context of physiological stress or inflammation. In fact, hypoalbuminemia is often an altered parameter observed in CanL dogs [33,34]. These contaminants probably diminished the capacity to detect more EVs-associated proteins. In fact, 32% of the protein identifications were associated with 1 UP and therefore excluded from most analyses. Still, the presence of plasma contaminants did not prevent the detection of EVs-specific markers in each individual sample [30]. Most individual markers were not detected in all samples. This might be explained by the heterogeneity of the cohort. Still, Integrin alpha and beta were consistently identified in the individual samples, with ITGB3 being the most prevalent one. This integrin, mostly associated with platelets, has been recently involved in metastatic development in cancer [35]. The Heat shock protein family A (HSP70) member 8 (HSPA8) was also detected in most samples. Considering that no prior constraints on age, sex, or dietary conditions, the consistency of detection of markers such as ITGB3 is relevant. Unexpectedly, some markers were only consistently detected in one of the groups. The clearest example of EVs marker segregation was CD82, mostly found in healthy samples. This might indicate that EVs populations differ upon infection. A previous study analysing the tetraspanin expression in peripheral blood leukocytes from healthy controls and in patients with bacterial infections was altered [36]. Notably, the tetraspanin with the highest significant differences in expression was also CD82, since no expression in B cells was detected in the patients with infections. It is plausible that this change in cells’ tetraspanins expression will impact the vesicles in circulation. Moreover, CD82 has been shown as an important player in phagocyte infiltration restraining and controlling inflammation. CD82 knockout macrophages lead to larger lesions upon *L. mexicana* infection, demonstrating the role of this tetraspanin in infection control [37]. Furthermore, when analysing the abundance of classical tetraspanins in the quantitative analysis, it was observed that CD9 and CD81 are less abundant in the CanL group. Overall, the modification of most relevant EVs markers observation can have a significant impact on future studies, especially considering positive selection approaches. The CD5L was also identified in all preparations. It is described by MISEV2018 as a secreted protein recovered with EVs; it was also described as an exosomal marker of plasma-derived EVs in human samples [27]. Although the abundance ratio of CD5L was not significantly different in both groups, it was previously found to be increased in patients with lung cancer, with its expression correlated to cancer tissues, and CD5L was suggested as a non-invasive biomarker for this type of cancer [38]. CD5L has been shown to have a role in several pathologies, mostly inflammatory diseases, ranging from infections to obesity or cancer [39]. Moreover, a 10-fold increase in CD5L plasma levels has been described 3 weeks after murine infection with *M. tuberculosis* [40]. Similar to what was observed in albumin and CD82, several proteins were either enriched or detected in one specific group. The GO analysis of core proteins revealed that these proteins were responsible for most of the GO term enrichment detected in the individual groups. In fact, the GO enrichment analysis revealed 20 biological processes associated with the core proteome, which matched perfectly the GO terms enriched in the CanL group. Most GO enrichment deviations from the core proteome were associated to the Healthy group, with 12 GO terms enriched only in the Healthy dataset. Most Healthy-specific GO enrichment was associated with generic biological processes, such as endocytic recycling, Golgi organization and protein transportation. The lack of enrichment of these GO terms in the CanL group might be related to a shift from homeostatic conditions caused by the infection. Considering the CanL-specific GO enrichment, only the antigen processing and presentation of peptide antigen via MHC class I (GO:00002474) was observed. Moreover, the cellular compartment GO associated with the MHC class I protein complex was enriched. A possible cause for this enrichment might be the diminution of EVs associated with red blood cells. This is supported not only by the clinical evidence of anaemia, but also by the decreased detection of red-blood-cells-related proteins. Still, it is intriguing that this was the only biological process that was significantly more represented in the CanL group. Nonetheless, MHC class I presentation through exosomes has been observed in other pathogens. For example, *M. tuberculosis* antigens presentation through infected cells exosomes can function as an alternative cross-presentation mechanism to induce acquired immune response. These exosomes activate CD8 T-cells, indicating that these antigens can be processed through MHC class I presentation pathways, and can help mount the CD8 T-cell response upon *M. tuberculosis* infection [41]. Therefore, a similar biological phenomenon might happen in the context of *Leishmania* infection, especially because, as with *M. tuberculosis, Leishmania* is also an intracellular pathogen that infects macrophages. Furthermore, also upon *H. pylori* infection, exosomal miR-155 released from infected macrophages promotes cytokine production to regulate inflammatory response that consequently leads to an expression of cellular signal transduction proteins, among them MHC-I, to regulate the immune response [42]. However, the downregulation of MHC-I is a common mechanism of different pathogens to invade the host. In human herpesvirus-6, this might be achieved through the transfer of MHC-I and MHC-II to exosomes [43]. A similar immune evasion mechanism mediated by the release of EVs could also justify the increased detection of EVs associated with MHC-I in CanL. Two other CanL-specific Molecular function GO-related observations were noteworthy, the enrichment of calcium ion binding and serine-type endopeptidase inhibitor activity. Phagocytes intracellular calcium concentration was shown to be increased upon *Leishmania* infection, probably as a response to a transient decrease of intracellular calcium storage upon infection [44]. This enrichment in the frequency of calcium ion binding proteins might be related to enhanced signalling needs in response to infection. Concerning the identification of serine-type endopeptidase inhibitor GO enrichment, it should be highlighted that in the unique healthy serine-type endopeptidase activity is enriched. Serine endopeptidase inhibitors are a protein superfamily associated with anticoagulant properties [45]. Thus, serine-type endopeptidase inhibitor activity GO term in CanL dogs might be associated with the prevention of coagulation processes. In fact, disseminated intravascular coagulation was previously observed in dogs infected with *L. infantum* [46]. Furthermore, the presence of circulating immunocomplexes, frequently observed in diseased dogs, activates coagulation pathways in the context of visceral leishmaniasis [47,48]. Thus, a possible mechanism of protection against these harmful coagulation events might be an increased presence of proteins that prevent coagulation. Nonetheless, the relative abundance of the individual proteins associated with these GO terms was not significantly increased in CanL. The detection of group-specific proteins was further exploited by a qualitative analysis through the identification of spectra in individual runs. This allowed the identification of two particularly interesting proteins, Myo-inositol oxygenase, detected in 44 out of the 48 injections associated with the Healthy group; and carboxylesterase 5 A, detected in 40 out of the 52 injections of the CanL group and in only 4 Healthy injections [10]. Myo-inositol oxygenase is a renal proximal tubular–specific enzyme, that catalyzes the oxidation of Myo-inositol to D-glucuronate, being considered an essential enzyme for inositol metabolism. The upregulation of this enzyme in the kidney is associated with increased production of inflammatory cytokines and ROS [49]. The plasma levels in humans were associated with the progression of chronic kidney disease [50]. Still, the biological implications of the reduced detection of Myo-inositol oxygenase are not evident because kidney damage is a frequent clinical sign in CanL dogs. Interestingly, increased Myo-inositol, the natural substrate of MIOX can cause in vitro depolarization of macrophages, altering the phagocytic potential of macrophages. This was shown in the context of antibiotic-resistant *E. coli.* [51]. Carboxylesterase 5 A can be associated with xenobiotic metabolism [52]. It is highly secreted in cats being found in the urine of these animals. We cannot exclude that the diseased animals have increased circulating carboxylesterase 5A associated with veterinary medical interventions with drugs. Moreover, reports on naturally infected dogs demonstrated changes in plasma concentration of lipids, such as cholesterol. These changes might be associated with hepatic disorders caused by infection and the deposition of immunocomplexes. The presence of carboxylesterase 5A associated with the EVs fractions recovered in diseased animals might be a sign of these perturbations. 

The qualitative approach, although invaluable, does not convene the full biological picture associated with the EVs preparations. Thus, a quantitative approach comparing the Healthy group with the CanL was performed. The reduced number of proteins with significantly altered abundance might be due to the already mentioned cohort heterogeneity and the significant amount of non-EVs contaminants. None of the proteins identified as more abundant in CanL presented a high PSM count, thus, the real biological relevance is difficult to ascertain. Concerning the proteins significantly less abundant in CanL EVs, several had a high number of PSMs. Three of them overlapped with the 10 most abundant proteins in the merge of the healthy samples—GLOBIN domain-containing protein, protein 4.1 and haemoglobin subunit alpha. Among the other proteins significantly less abundant in CanL with a detection profile with more than 2 UP and more than 50 PSMs carbonic anhydrase, biliverdin reductase B and glycophorin C were mostly identified in the Healthy group. GO assessment of the proteins revealed that the terms enriched are associated with red blood cell biology. These observations were in agreement with previous studies that report a haemoglobin downregulation in the context of infection [53]. It has been reported that inflammation associated with CanL can cause anaemia and consequently prevent the body from using the stored iron reservoirs, which may cause a decrease in haemoglobin. Decreases in haemoglobin levels were verified in different situations. Among them, haemoglobin subunit beta levels were downregulated in dogs’ serum after treatment with anti-*Leishmania* drugs [54]. The downregulation of haemoglobin subunit-α has previously been described in *Leishmania-panamensis*-infected human skin lesions [55]. Haemoglobin and globin, together with the carbonic anhydrase, another protein also significantly less abundant in the CanL group, were also downregulated in the saliva of experimentally *L. infantum* infected dogs with clinical signs [53], which is in agreement with the findings made in CanL EVs. Anaemia can be caused by chronic renal failure, haemorrhage, haemolysis, bone marrow hypoplasia or aplasia and decreased erythrocyte membrane lipid fluidity [56,57,58]. Infection can also impact erythropoiesis due to changes in the bone marrow [59] and kidneys [56]. CanL dogs frequently present alterations in the erythrogram status. Moreover, studies have described a correlation between the clinical signs and severity of anaemia [60,61,62]. In fact, 6 out of the 20 CanL dogs had anaemia, which corroborates with the results obtained in this study. It has also been shown that CanL-associated anaemia can result from impaired erythrocyte membrane fluidity [57]. Other less abundant proteins, such as protein 4.1, anion exchange protein, and erythrocyte membrane protein 4.2 are all integrant parts of the membrane skeleton of erythrocytes. Rh protein, glycophorin A, and glycophorin C are all proteins present in the red blood cell membrane. In fact, alterations in glycophorin C, a protein that interacts with the erythrocytes cytoskeleton, are involved in hematopoietic homeostasis [63]. Erythrocytes membrane structural integrity is necessary for their function. Thus, perturbations of the membrane characteristics, such as fluidity, oxidative changes, or ligand-specific interactions often result in pathology [64]. Moreover, the aforementioned carbonic anhydrases were shown to be also diminished in dogs’ saliva in CanL dogs [53] and have antimicrobial activity and contribute to the maintenance of pH homeostasis in the mouth [65]. The downregulation of this protein was suggested to be an organism’s response against the parasite by diminishing antioxidant compounds. Few proteomic studies analysing dogs with CanL are reported [53,66,67], two of which analysed canine serum, one of them from infected dogs and healthy dogs [67] and the second one compared the proteins from asymptomatic and symptomatic stages of infection [66]. None of the differentially abundant proteins found in these studies were significantly enriched in our samples.

Although the samples chosen in this study present variability in different parameters such as age and breed, it was possible to identify possible EVs-associated proteins such as carbonic anhydrase, biliverdin reductase B and glycophorin C that were consistently detected in the Healthy animals.

The identification of proteins associated with *L. infantum* would be of high value not only for a better understanding of the infection but also as possible exploitable biomarkers. However, only one unique peptide was identified for each protein and most proteins only appeared in one sample. This can be explained by the presence of other highly abundant peptides from the host that prevent the detection of multiple peptides from the same protein. That was also observed for *T. cruzi* when trying to identify biomarkers from plasma-derived EVs in a heart transplant patient with chronic Chagas disease [17]. Nonetheless, when comparing the PSMs/UP associated with CanL and Healthy groups when using databases from two other pathogens, *N. caninum* and *P. falciparum,* the data suggests *Leishmania*-specific protein identification. Interestingly, the PSMs/UP ratio for the Healthy animals obtained using the *Leishmania* database was similar to the one obtained for the other pathogens, further strengthening the *Leishmania*-specific identifications for the CanL group. 

Another limitation of the study was the possibility of subclinical infections. We tried to limit this by using also animals from non-endemic regions, but we cannot exclude that some negative identifications might be indeed *Leishmania*-specific.

Among the parasite proteins identified in more than one sample, putative DNA Polymerase epsilon subunit b was the one most consistently detected being detected in six dogs. The catalytic subunit of this protein was previously identified in circulating immunocomplexes of VL patients [25]. A significant difference in seroreactivity was verified when the Healthy group and the CanL group were compared suggesting that the protein is present during the infection. Significantly, DNA Polymerase epsilon subunit b peptides were identified in five seropositive animals.

Overall, this study is the first characterization and proteomic analysis of EVs recovered from dogs’ plasma. The core proteome of this type of EVs was described and changes associated with CanL were identified. It was established that EVs can be useful to understand the pathology since several known *Leishmania*-specific haematological and biochemical alterations were also possible to associate with the EV molecular cargo. In addition, some intriguing protein identification such as Myo-inositol and carboxylesterase 5 A might be proteins of interest for CanL management that were previously unknown. Interestingly, it was possible to find with some consistency some parasite-specific proteins. Among these, the most relevant was the putative DNA polymerase epsilon subunit b. The value of these identifications is still uncertain because they are not supported by more than one UP. Further experiments with dogs afflicted with other pathologies are needed to evaluate if the markers found are adequate for CanL management. The presented data paved the way for canine plasma EV studies and enables inter-species comparisons. 

## 4. Materials and Methods

### 4.1. Parasites and Cell Culture

*L. infantum* (MHOM/MA/67/ITMAP-263) promastigotes were maintained in standard RPMI 1640 medium supplemented with 10% Fetal Bovine Serum (FBS), 2 mM L-glutamine, 100 U/mL penicillin, 100 mg/mL streptomycin and 20 mM HEPES buffer (all products from Lonza, Basel, Switzerland) at 26 °C. Cultures were grown with a starting inoculum of 1 × 10^6^ parasites. 

### 4.2. Antigens

For SPLA production, 5 days old promastigotes were washed three times with PBS and centrifuged at 3500× *g*, 10 min, at 4 °C. The pellet was suspended in PBS containing 1 mM phenylmethylsulfonyl fluoride (PMSF) protease inhibitor and submitted to 10 freeze–thaw cycles for the parasites’ rupture. The suspension was centrifuged at 13,000× *g*, 30 min, at 4 °C and the supernatant was recovered, quantified by DC (detergent compatible) protein assay (Bio-Rad Laboratories, Hercules, CA, USA), and stored at −80 °C in single aliquots. 

The rK39, obtained from Dr Steven Reed (Infectious Disease Research Institute, Seattle, WA, USA), was suspended H2O, quantified and stored at −80 °C in single-use aliquots.

### 4.3. Collection, Characterization, and Selection of Biological Samples

During this study, thirty-nine samples were selected (20 CanL dogs and 19 healthy dogs). The 20 CanL dogs were from continental Portugal in regions endemic to *L. infantum* infection. Healthy samples 1–11 were from regions without known active *Leishmania* transmission, Azores, and Ireland, the remaining 9 were from endemic areas. Peripheral blood was collected by venepuncture in EDTA K3 tubes (Sarstedt, Sarstedtstraße, Germany). Samples were centrifuged at 400× *g* for 10 min at room temperature. Plasma was collected and posteriorly centrifuged twice at 2000× *g* for 10 min at 4 °C. Supernatant was recovered and frozen at −80 °C. Dogs were included in CanL group if they were polysymptomatic for CanL and presented at least two of the three criteria (1) seropositive for at least one *Leishmania-*specific antigens (SPLA and rK39) by the in-house ELISA; (2) positive result on a rapid-chromatographic test validated for CanL; (3) positive parasite culture from blood, lymph node or bone marrow cells seeded in Schneider media. Healthy dog samples were obtained from animals fulfilling the criteria to become eligible for blood donation programs. All healthy dogs met the following criteria: (1) absence of clinical evidence of disease; (2) seronegative results for *Leishmania*-specific antigens (SPLA and rK39) by ELISA and commercially available rapid chromatographic tests validated for CanL diagnosis; (3) negative *Leishmania* blood PCR. 

### 4.4. EVs Separation

EVs were isolated from canine plasma samples by size-exclusion chromatography (SEC) as previously described by de Menezes-Neto et al., 2015 [27], with some modifications. Aliquots of plasma were thawed on ice and processed by centrifugation at 2000× *g* for 10 min at 4 °C. One hundred microliters of supernatant were then loaded on the top of a 1 mL Sepharose CL-2B (Cytiva, Marlborough, MA, USA) column pre-equilibrated with PBS. Ten fractions of 100 µL were collected immediately after sample loading and frozen at −80 °C. A small aliquot of each was kept to measure protein concentration by BCA (Thermo Scientific, Waltham, MA, USA) by measuring absorbance an automatic reader (Synergy2, BioTek, Winooski, VT, USA) and to perform the bead-based assay. This procedure was repeated at least 3 times, using new columns for a total volume of processed plasma of 300 µL minimum (100 µL/column).

### 4.5. Bead-Based Flow Cytometry 

The bead-based flow cytometry assay was performed based on conjugation of latex microbeads to EVs followed by antibody coupling for FACS analysis [68]. The EVs obtained from canine plasma, and isolated by SEC, were coupled to latex microbeads and then incubated with antibodies to detect the presence of at least two of the three EVs markers, CD9, CD5L [27] and CD71 [28,29], in the preparations. Briefly, 50 µL of the SEC fractions were coupled to Aldehyde/Sulfate Latex Beads, 4% *w/v*, 4 µm (Invitrogen, Waltham, MA, USA) by incubation of 15 min, with agitation every 5 min. Coupled beads were then blocked with 1 mL of BCB Buffer (PBS 1X/BSA 0.1%/NaN3 0.01%—from Sigma-Aldrich, St. Louis, MO, USA), incubating overnight in a rotation device. Beads were further centrifuged at 2000× *g* for 10 min, the supernatant was removed, and the pelleted beads were suspended in 150 µL of BCB buffer. 45 µL of bead suspension was incubated with anti-CD5L antibody (Abcam: ab45408, Cambridge, UK) at 1:5000 dilution, anti-CD71 antibody (Abcam: ab84036) at 1:1000 dilution or anti-CD9 (Immunostep 9PU-01MG, Salamanca, Spain) at 1:500 dilution for 30 min at 4 °C in a round bottom plastic microplate. After washing, samples previously incubated with CD9 were incubated with α-mouse secondary antibody conjugated to Alexa 488 (Invitrogen: A11001) and fractions incubated with CD5L and CD71 were incubated with α-rabbit secondary antibody conjugated to Alexa 488 (Invitrogen: A11008) both at 1:500 dilution for 30 min at 4 °C, protected from light. After two wash steps, the beads were suspended in 100 µL of PBS and analysed by flow cytometry using a BD FACSLyric flow cytometer. MFI and bead count data were obtained using FlowJo V10 Software (Tree Star, Woodburn, OR, USA). As a control for specificity, a pool of fraction 5 and 6 obtained from SEC coupled to beads was incubated with the secondary antibody Alexa 488 at dilution 1:500. 

### 4.6. Nanoparticle Track

Size mode determination (n = 8 for the Healthy group; n = 12 for the CanL group) was performed resorting to Nanoparticle Track Analysis (NTA) in a NanoSight LM1012 instrument (Malvern Instruments Ltd., Malvern, UK) using the NTA software (version 3.2). The instrument is equipped with a 638 nm laser, a system of video capture and a particle-tracking software. The fractions with highest fluorescence values to CD5L/CD71/CD9 were analysed in this equipment. Size determination was considered when the number of particles per frame was in a range from 10 to 120.

### 4.7. Transmission Electron Microscopy

Representative images of canine plasma-derived EVs recovered by SEC were obtained with negative staining transmission electron microscopy, using Jeol JEM 1400 transmission electron microscope (JEOL, Tokyo, Japan) and images were digitally recorded using a CCD digital camera Orius 1100W (Tokyo, Japan). Digital images of EVs from each sample were taken.

### 4.8. Proteomic Analysis and Protein Identification

After physical characterization of Evs, the proteomic analysis proceeded. Digestion of the samples (10–100 µg protein) was performed with trypsin/LysC (1:50) overnight following solid-phase-preparation (SP3) previously described [69]. The concentration of the resulting peptides was measured by fluorescence. The proteomic analysis of EVs was performed by injection of 500 ng of peptides in a nano LC (Ultimate 3000, Thermo Fisher Scientific, Bremen, Germany) connected to a Q Exactive Hybrid Quadrupole-Orbitrap mass spectrometer (Thermo Fisher Scientific, Bremen, Germany) as previously described [70]. The LC-MS/MS raw data was analysed by Proteome Discoverer 2.5.0.400 (Thermo Fisher Scientific). For protein identification, the proteomes from four taxonomic selections were considered, *Leishmania infantum* (8045 entries, 2019_11), *Canis lupus familiaris* (45,301 entries, 201_11), *Plasmodium falciparum* (129,297 entries, 2022_01) and *Neospora caninum* (6933 entries, 2022_02) from UniProt, together with a database of common contaminants from MaxQuant.

### 4.9. Data Analysis

Canine peptides were selected based on a maximum false discovery rate (FDR) of 1%. *Canis lupus familiaris* proteins identified only with one unique peptide were also excluded. Common contaminants were excluded from the analysis. The data for canine proteins was analysed using the merged data from the CanL dogs EVs and the merge data of the healthy dogs EVs. Moreover, for quantification purposes, the data was analysed using the data from both groups to perform the ratio of abundances (CanL/Healthy). The data was transformed into logarithmic scale (Log2). Subsequently, a statistical analysis was performed considering the ratio of abundances. Thus, a fold change and a correspondent *p*-value were reported for each protein. This data allowed the construction of a volcano plot that highlighted the upregulated and the downregulated proteins. Enrichment analysis of canine proteins with statistically different abundance in CanL dogs were performed with the Database for Annotation, Visualization, and Integrated Discovery (David 2021) [71]. 

*L. infantum* proteins were evaluated in an individual sample level. *L. infantum* proteins with one unique peptide identified were considered. Proteins identified in Healthy dogs EVs were considered false positives and were excluded from the analysis. All peptides identified were subjected to homology analysis in NCBI, and peptides only identified in *Leishmania* species were valued. Moreover, the proteins were only considered as potential candidates if they appeared in several individual samples.

### 4.10. DNA Isolation

*Leishmania infantum* (MHOM/MA/67/ITMAP-263) genomic DNA was extracted using DNAzol (Invitrogen, Waltham, MA, USA), following the manufacturer’s instructions. DNA quality and concentration were determined with NanoDrop (Thermo Fisher Scientific, lmington, DE, USA), and then stored at −20 °C until use.

### 4.11. Putative DNA Polymerase Epsilon Subunit b Cloning 

Fragments of the open reading frames of putative DNA Polymerase epsilon subunit b (LinJ.35.1780; chromosome LinJ.35; 666433-668091) were PCR-amplified, using primers 1 (5’ CATATGATGAGGGAGCCCAAGGATG 3’) and 2 (5’ GGATCCTTACTCCAGCTATTGAGGCTGC 3’) PCR conditions used for the amplification of putative DNA Polymerase epsilon subunit b were: initial denaturation (2 min at 94 °C), 10 cycles of denaturation (15 s at 94 °C), annealing (30 s at 53.8 °C,) elongation (2 min at 72 °C), followed by 20 additional cycles of denaturation (15 s at 94 °C), annealing (30 s at 53.8 °C,) elongation (2 min + 5 s per cycle at 72 °C) and a final extension step (7 min at 72 °C). All the PCR products were obtained using a Taq DNA Polymerase with proofreading activity (Roche, Basel, Switzerland). The fragments were isolated and cloned into pGEM-T Easy vector (Promega, Madison, WI, USA) and posteriorly sequenced. The putative DNA Polymerase epsilon subunit b genes were excised from the pGEM-T Easy vector using NdeI/BamHI, gel purified and subcloned into pET28a(+) expression vector (Novagen—Sigma-Aldrich, St. Louis, MO, USA). The resulting plasmids presented a hexa-histidine tag in the N-terminal and were transformed into *E. coli* BL21 DE3 cells (Invitrogen, Life Technologies, Carlsbad, CA, USA).

### 4.12. Protein Production 

The recombinant protein was grown in at least 1 L of LB broth medium supplemented with 5 g/L of NaCl and 50 µg/mL Kanamycin. Precultures were grown overnight at 37 °C. Cells were induced with 1 mM IPTG and were grown at 37 °C. After 4 h, the cells were harvested at 4 °C, maximum speed for 30 min. The pellet was stored at −20 °C. 

### 4.13. Inclusion Bodies Isolation and Solubilization

The pellet corresponding to 200 mL of culture was resuspended in 8 mL of resuspension buffer [20 mM Tris-HCl, pH 8.0]. Lysozyme was added to a final concentration of 0.2 mg/mL, as well as 10 μg/mL DNase and 1 mM MgCl_2_ and incubated 30 min on ice. The cells were disrupted with sonication using S-250A Model Sonifier Analog Cell Disrupter (Branson Ultrasonics, Brookfield, Connecticut, United States) using the following conditions: macrotip; 10 cycles; 10 pulses; output control 4; duty cycle 50%. A centrifugation step followed, and the supernatant was discarded. The pellet was suspended in 6 mL of cold isolation buffer [20 mM Tris-HCl, pH 8.0; 2 M Urea; 0.5 M NaCl; 2% Triton-X 100]. A sonication step followed with the conditions: microtip; 5 cycles; 10 pulses; output control 3; duty cycle 50%. A centrifugation was performed, the supernatant was removed and the pellet resuspend pellet in cold isolation buffer. The sonication, centrifugation and resuspension steps were repeated two more times, in the same conditions. The remaining pellet was stored at −20 °C for the solubilization protocol. The pellets were resuspended in 10 mL of 8 M urea buffer [20 mM Tris-HCl pH 8.0; 0.5 M NaCl; 8 M Urea; 1 mM β-mercaptoethanol] and were incubated for 1 h at room temperature on the rotation device. A centrifugation was performed to remove any particles and the protocol was completed with a filtration with a 0.45 μm filter (Millipore, Merck, Burlington, MA, USA). The sample was kept to use in the preparative SDS-PAGE protocol.

### 4.14. Semi-Quantification

To determine the concentration of putative DNA Polymerase epsilon subunit b, prior and post protein extraction protocol, SDS-PAGE analyses were performed, using bovine serum albumin (BSA) as a standard. ImageJ software (ImageJ, National Institutes of Health) was used to determine the mean gray values through the intensity of the bands. The calibration curve was determined by plotting the mean gray value (MGV) against BSA mass. 

### 4.15. Preparative Gels and Protein Extraction

Four 10% (*m*/*v*) poliacrilamide 1.5 mm SDS-PAGE gel were prepared and loaded with 390 µg of putative DNA Polymerase epsilon subunit b. The gels were run with an upper buffer [Tris/Glycine + 0.05% SDS + 25 mg/L Coomassie R250], to avoid posterior staining. The bands of interest were cut and put in a single 50 mL falcon tube. 6 mL of protein extraction buffer [2 mM DTT, 0.5 mM PMSF, 0.02% SDS] was added to gel. The gel was homogenized into small beads with a polytron and incubated overnight at 4 °C on rotation. A centrifugation at 4000× *g* followed and the supernatant was kept. Another round of protein extraction succeeded (incubation overnight at 4 °C and supernatant recovery). The supernatants were frozen at −80 °C and posteriorly lyophilized. The protein was suspended in 1 mL of water, and cold acetone in a 4:1 ratio was added. The falcon was frozen overnight, and the protein was pelleted the next day at 4000 g for 30 min. The pellet was washed with ice cold 90% acetone, centrifuged and dried at 37 °C. Finally, the protein was resuspended in PBS and homogenized with weak sonication.

### 4.16. Enzyme-Linked Immunosorbent Assay

Flat-bottomed 96 well high-binding microtiter plates (Greiner, Kremsmünster, Austria) were coated with 5 µg/mL of recombinant proteins rK39 and putative DNA Polymerase epsilon subunit b; or 10 µg/mL of soluble promastigote *Leishmania* antigens. All antigens/extracts were diluted in carbonate buffer [0.04 M NaHCO_3_, 0.01 M Na_2_CO_3_, pH = 9.6] and dispensed 50 µL/well. The plates were incubated overnight at 4 °C. Posteriorly, the plates were washed several times with PBS-Tween (0.05%) and blocked with PBS-low-fat-milk (3%), for 1 h at 37 °C. The plates were washed with PBS-Tween (0.05%) and canine sera were diluted (1:1500) in PBS-Tween (0.05%) and incubated for 30 min at 37 °C. After washing the plates, 30 min at 37 °C incubation with the secondary antibody, in the dark, occurred. α-dog IgG conjugated to horseradish peroxidase (A6792; Sigma, St. Louis, Missouri, United States) was diluted 1:1500 (for the rK39 and SPLA) or 1:1000 (for putative DNA Polymerase epsilon subunit B) in PBS-Tween (0.05%). The plates were washed and developed for 10 min in the dark at room temperature (RT) using 0.5 mg/mL ο-phenylenediamine (OPD; Sigma, St. Louis, Missouri, United States) in citrate buffer [0.05 M C_6_H_8_O_7_.H_2_O; 0.024 M Na_2_HPO_4_; pH = 5.3] as substrate and hydrogen peroxide as reactive oxygen metabolic by-product. Reaction was stopped with HCl 3 M and absorbance values were read at 492 nm in a Synergy 2 automatic reader (BioTek, Winooski, VT, USA).

### 4.17. Statistical Analysis

The cut-offs for SPLA and rk39 were calculated elsewhere [72]. 

To determine the seropositivity for the putative DNA Polymerase epsilon subunit b, a cut-off was determined using the Healthy group. The cut-off was calculated as the mean + 2 standard deviations of the ODs from the Healthy group.

All statistical analysis in Figure 1, Figure 2 and Figure 9 was performed using GraphPad Prism software (Version 9.4.0).

## Figures and Tables

**Table 1:** Characterization of the Healthy and CanL groups.

	Sample Code	Location	Age	Gender	Breed	Indirect Parasitological Diagnosis (Rapid Chromatographic Test or in-House ELISA)	Direct Parasitological Diagnosis (Blood PCR or Culture)
**Healthy**	1	Azores, Portugal	Puppy	Male	Mongrel	negative	negative
	2	Azores, Portugal	Adult	Male	Labrador Retriever	negative	negative
	3	Azores, Portugal	Puppy	Female	Mongrel	negative	negative
	4	Ireland	Adult	NA	UN	negative	negative
	5	Ireland	Adult	NA	UN	negative	negative
	6	Ireland	Adult	NA	UN	negative	negative
	7	Ireland	Adult	NA	UN	negative	negative
	8	Ireland	Adult	NA	UN	negative	negative
	9	Ireland	Adult	NA	UN	negative	negative
	10	Ireland	Adult	NA	UN	negative	negative
	11	Ireland	Adult	NA	UN	negative	negative
	12	Braga, Portugal	Adult	Male	Greyhound	negative	negative
	13	Porto, Portugal	Adult	Male	Greyhound	negative	negative
	14	Braga, Portugal	Adult	Male	Greyhound	negative	negative
	15	Braga, Portugal	Adult	Male	Greyhound	negative	negative
	16	Porto, Portugal	Adult	NA	UN	negative	negative
	17	Porto, Portugal	Adult	Female	UN	negative	negative
	18	Braga, Portugal	Adult	Female	Greyhound	negative	negative
	19	Porto, Portugal	Adult	NA	UN	negative	negative
**CanL**	20	Bragança, Portugal	Adult	Male	Jack Russel Terrier	positive	NA
	21	Bragança, Portugal	Puppy	Female	Mongrel	positive	NA
	22	Bragança, Portugal	Young adult	Female	*Cão de Gado Transmontano*	positive	NA
	23	Bragança, Portugal	Young adult	Female	German Shorthaired pointer dog	positive	NA
	24	Coimbra, Portugal	Adult	Male	Mongrel	positive	positive
	25	Coimbra, Portugal	Adult	Male	Mongrel	positive	positive
	26	Bragança, Portugal	Adult	Male	Yorkshire Terrier	positive	NA
	27	Guarda, Portugal	Adult	Female	Jack Russel Terrier	positive	positive
	28	Castelo Branco, Portugal	Adult	Male	Mongrel	positive	positive
	29	Beja, Portugal	Young adult	Female	Mongrel	positive	positive
	30	Lisboa, Portugal	Senior	Male	Irish Setter	positive	positive
	31	Coimbra, Portugal	Mature	Male	Mongrel	positive	positive
	32	Castelo Branco, Portugal	Adult	Male	Mongrel	positive	positive
	33	Castelo Branco, Portugal	Adult	Female	Portuguese Podengo	positive	positive
	34	Coimbra, Portugal	Adult	Male	Portuguese Podengo	positive	positive
	35	Coimbra, Portugal	Adult	Male	Mongrel	positive	positive
	36	Santarém, Portugal	Young adult	Female	Beagle	positive	positive
	37	Porto, Portugal	Adult	Female	Portuguese Podengo	positive	positive
	38	Évora, Portugal	Adult	Female	German Shepherd mix	positive	positive
	39	Évora, Portugal	Adult	Male	Mongrel	positive	positive

NA—Not Available; UN—Undefined; Puppy—up to 1 year; Young adult—between 1 and 3 years; Adult—between 3 and 8 years; Mature—between 8 and 12 years; Senior > 12 years.

## Data Availability

The mass spectrometry proteomics data have been deposited to the ProteomeXchange Consortium via the PRIDE [73] partner repository with the dataset identifier PXD039610. Reviewer account details for PRIDE server: Username: reviewer_pxd039610@ebi.ac.uk; Password: uHp1Wg6q.

## Supplementary Materials

The following supporting information can be downloaded at: https://www.mdpi.com/article/10.3390/ijms24065490/s1.
