# Supplementary material for: Characterization and Proteomic Analysis of Plasma EVs Recovered from Healthy and Diseased Dogs with Canine Leishmaniosis
Source: Int J Mol Sci. 2023 Mar 13;24(6):5490. doi: 10.3390/ijms24065490 (PMC10056832; doi:10.3390/ijms24065490)
Supplement: Supplementary file 1 [file ijms-24-05490-s001.zip › ijms-2208855-supplementary/Captions for tables S1, S2, S3, S4 and S6.pdf]

Table S1. Canine proteins identified in each sample. Each sheet in the Excel document corresponds to a different sample (samples 1 to 19—healthy samples; 20 to 39—CanL samples). Each protein is associated with the Protein FDR Confidence, Uniprot accession number, peptides, PSMs and unique peptides.

Table S2. Canine proteins that were identified in each group. Each sheet in the Excel document corresponds to a different group: Healthy, CanL, core proteome, Unique to Healthy and Unique to CanL. The different proteins are associated with the Uniprot accession number, peptides, PSMs and unique peptides.

Table S3. Gene ontology (GO) enrichment analysis of the proteins present in different groups. Each sheet in the Excel document corresponds to a different group: Healthy, CanL, core proteome, Unique to Healthy and Unique to CanL. GO enrichment analysis shows terms significantly enriched for biological processes, molecular function and cellular compartment, corresponding genes to each GO term and respective *p*-value.

Table S4. List of proteins identified in the injection analysis. Confidence associated with the peptide identification, Uniprot accession number and respective peptides, PSMs and unique peptides identified in each protein. Abundance ratio (CanL/Healthy) e respective *p*-value. The presence of green (Healthy) or red (CanL group) colours associated with individual proteins represents the detection of that PSM in the injection (corresponding to a technical replicate). Number of injections associated with Healthy group, CanL group, total number of injections and difference between the two groups.

Table S6. List of *L. infantum* proteins identified in CanL dogs. Respective TriTrypDB and Uniprot accession number, description, peptide sequence identified and peptide blast result performed in NCBI as well as peptides, PSMs, unique peptides and the total number of dogs associated with each protein.
