# Supplementary material for: Characterization and Proteomic Analysis of Plasma EVs Recovered from Healthy and Diseased Dogs with Canine Leishmaniosis
Source: Int J Mol Sci. 2023 Mar 13;24(6):5490. doi: 10.3390/ijms24065490 (PMC10056832; doi:10.3390/ijms24065490)
Supplement: Supplementary file 1 [file ijms-24-05490-s001.zip › ijms-2208855-supplementary/ijms-2208855-supplementary materials.pdf]

Supplementary Material

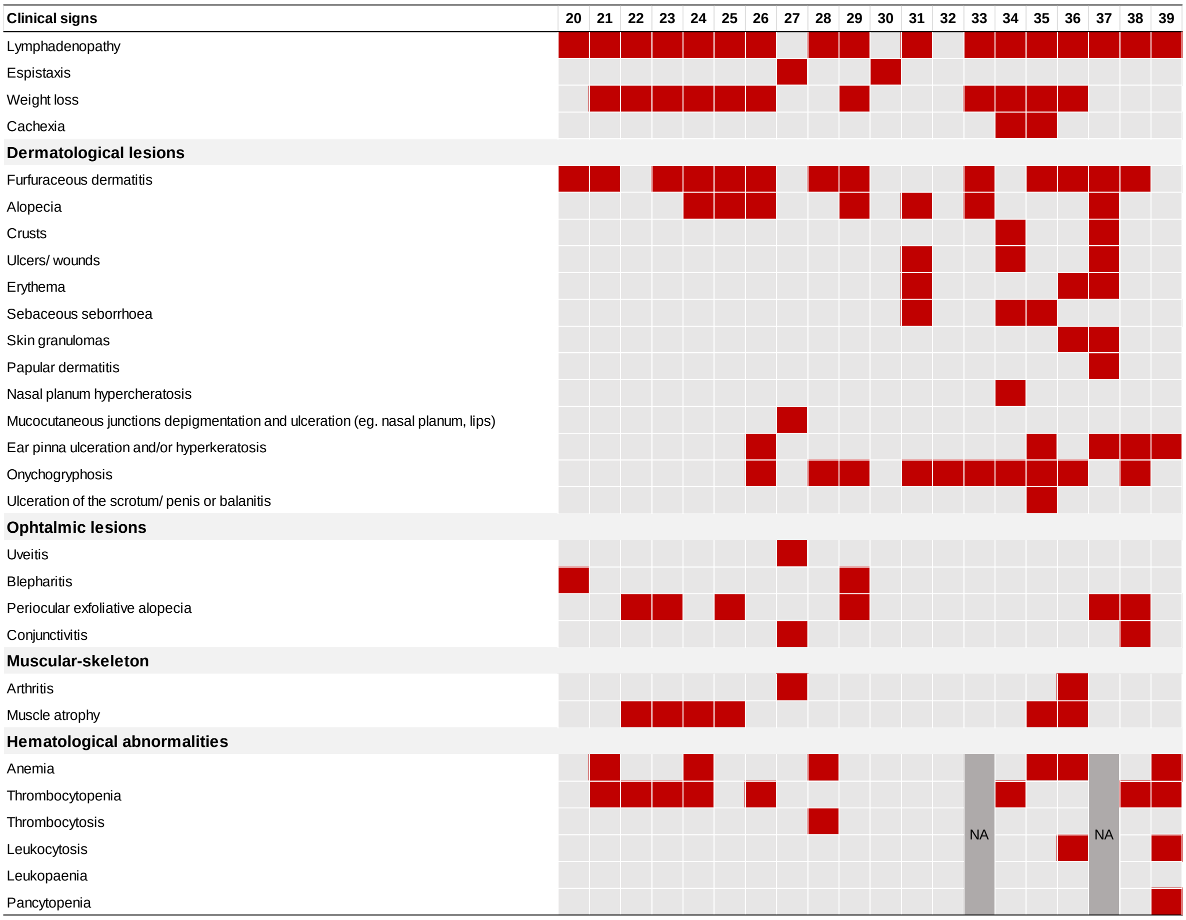

Figure S1. Clinical signs of the CanL dogs. Clinical signs are divided into unspecific, more general clinical signs (lyphadenopathy, epistaxis, weight loss and cachexia) and more specific clinical signs compatible with CanL – dermatological lesions, ophthalmological lesions, muscular skeleton lesions and hematological abnormalities. NA – not available.

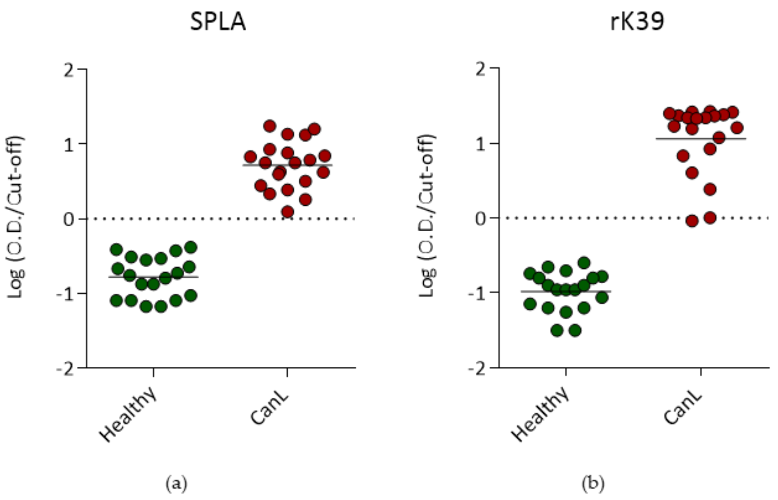

Figure S2. Reactivity of sera from CanL confirmed cases and healthy dogs to (a) SPLA and (b) rK39 determined by ELISA. Coatings were done with 10 µg/mL for SPLA and 5 µg/mL rK39. Results are represented as the logarithm of the optical density at 492 nm normalized by the cut-off value for each antigen.

The ashed lines represent the cut-off. Data was generated from the average of two independent assays.

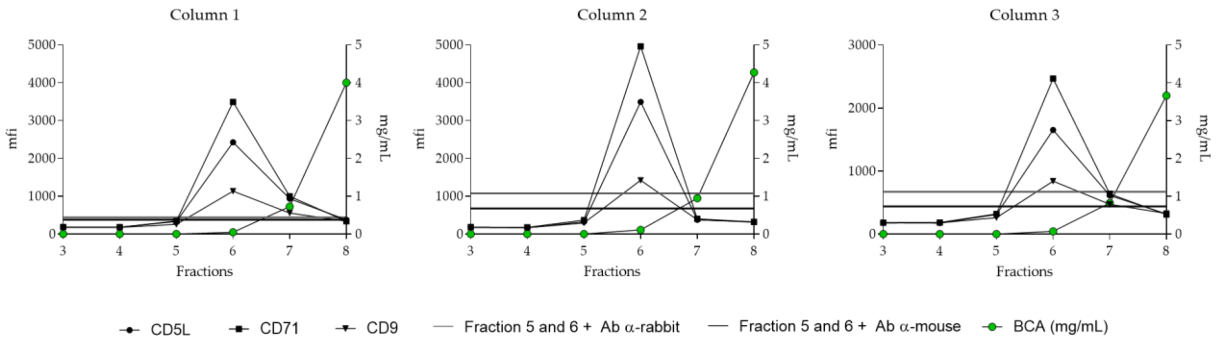

Figure S3. Results of BCA (mg/mL), bead-based assay for FACS analysis with CD5L, CD71 and CD9 and respective controls (mfi) for fractions 3-8 obtained from SEC for column 1, 2 and 3. Control – beads + Fraction 5 and 6 + secondary antibody.

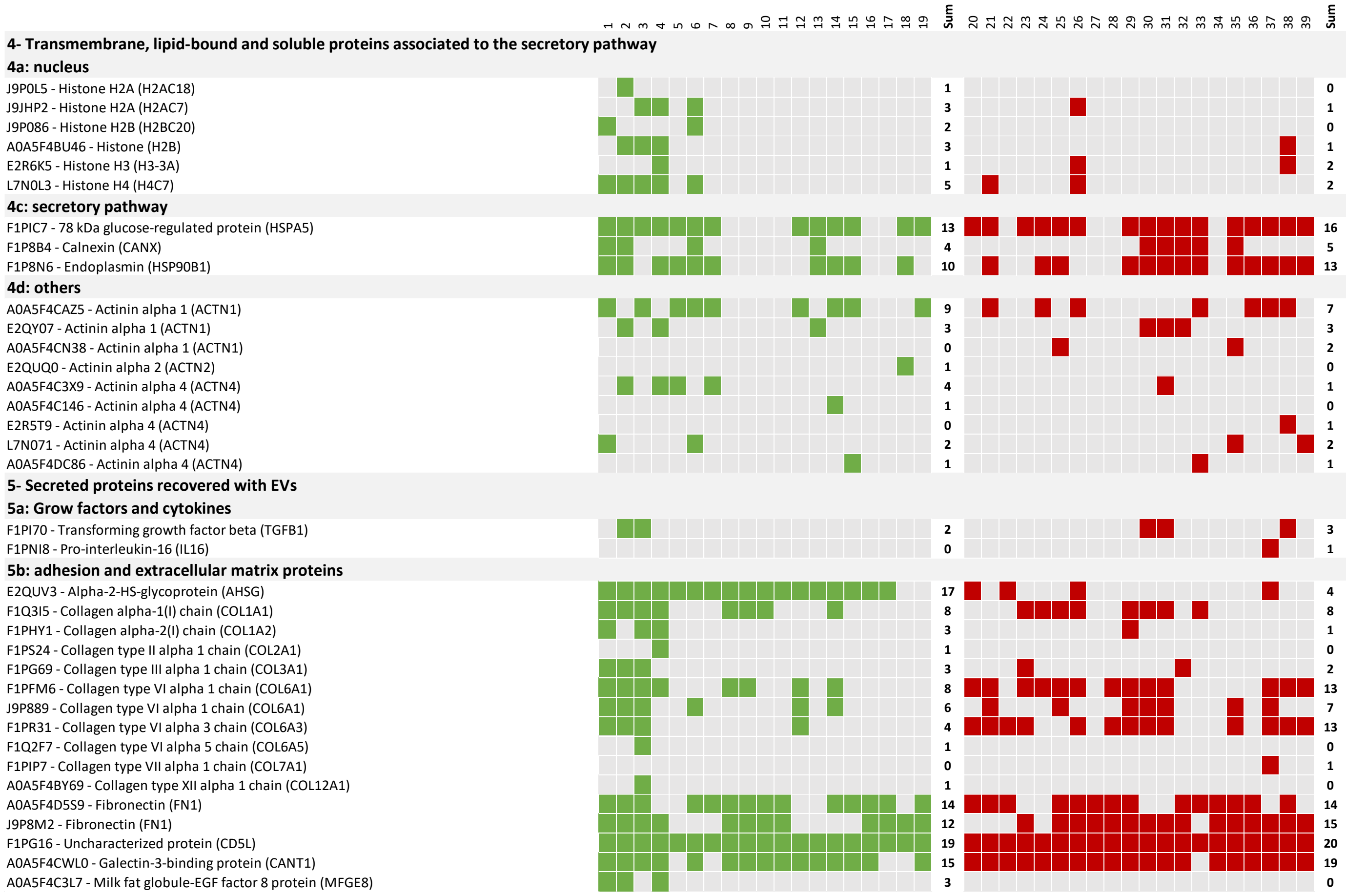

Figure S4. Protein content- EV characterization based on the Mysev2018. Each line represents a protein, while the columns labelled with a number represent the individual samples. The presence of green (Healthy group) or red colour (CanL group) associated to individual proteins depicted in lines represents the detection of that protein in the sample with at least 2 unique peptides.

Table S5. List of acession numbers, description, fold change and p value associated to the proteins significantly altered abundance in CanL EVs and respective peptides, Peptide-Spectrum Matches (PSMs) and unique peptides identified in healthy and CanL EVs.

Accession	Description	Fold change (CanL / Healthy)	p value	Peptides	PSMs	Unique Peptides
**Proteins significantly more abundant in CanL EVs**						
E2RSJ3	LIM domain containing preferred translocation partner in lipoma (SDF4)	100	1.7E-16	2	10	2
F1P9P7	Stromal cell derived factor 4 (SDHA)	100	1.7E-16	2	6	2
A0A5F4CYV2	Succinate dehydrogenase complex flavoprotein subunit A (LPP)	100	1.7E-16	2	2	2
F1PPE5	Biorientation of chromosomes in cell division 1 like 1 (BOD1L1)	100	1.7E-16	2	2	2
F1PRL5	Solute carrier family 25 member 5 (SLC25A5)	94.20	4.7E-05	2	2	2
**Proteins significantly less abundant in CanL EVs**						
F2Z4Q6	Serum albumin (AFP)	0.061	4.4E-02	61	30291	4
Q2Z1P8	Anion exchange protein (SLC4A1)	0.06	4.1E-02	35	5705	35
P60524	Hemoglobin subunit beta (HBB)	0.058	3.7E-02	17	4571	3
F1PYZ1	GLOBIN domain-containing protein (LOC609402)	0.058	3.8E-02	18	4207	2
P60529	Hemoglobin subunit alpha (HBA)	0.055	3.1E-02	11	2110	2
Q6Q7P4	Protein 4.1 (EPB41)	0.045	1.6E-02	34	1350	4
F1PD51	Erythrocyte membrane protein band 4.2 (EPB42)	0.042	1.3E-02	33	934	32
E2QVU9	Biliverdin reductase B (BLVRB)	0.038	9.1E-03	11	490	11
F1PBK6	Carbonic anhydrase 1 (CA1)	0.038	9.2E-03	13	431	13
F1PDY8	Carbonic anhydrase 2	0.036	7.8E-03	14	350	14
A0A5F4DER9	Aquaporin-1 (AQP1)	0.036	7.9E-03	4	217	4
A0A5F4D567	Uncharacterized protein	0.036	1.3E-02	9	208	2
A7VLI5	Glycophorin C (GYPC)	0.034	5.8E-03	3	202	3
Q3BCQ5	Ammonium transporter Rh type A (RHAG)	0.028	8.6E-04	4	123	4
Q3BCQ6	Rh antigen-like protein (Rh30)	0.028	2.8E-03	4	93	4
P02727	Glycophorin-A (GYPA)	0.027	2.4E-03	2	51	2
E2R629	Atypical chemokine receptor 1 (ACKR1)	0.026	2.2E-03	2	35	2
E2QTD8	Myo-inositol oxygenase (MIOX)	0.017	3.1E-04	3	15	3
F1PBM2	Vesicle trafficking 1 (VTA1)	0.010	1.7E-16	2	9	2
A0A5F4DD49	Serine incorporator 3 (SERINC3)	0.010	1.7E-16	2	7	2
P27597	T-cell surface glycoprotein CD3 epsilon chain (CD3E)	0.010	1.7E-16	3	5	3
A0A5F4CCI6	Serine/threonine kinase 24 (STK24)	0.010	1.7E-16	2	5	2
E2RK13	Protein XRP2 (RP2)	0.010	1.7E-16	2	5	2
E2R3J3	G protein subunit alpha z (GNAZ)	0.010	1.7E-16	2	3	2

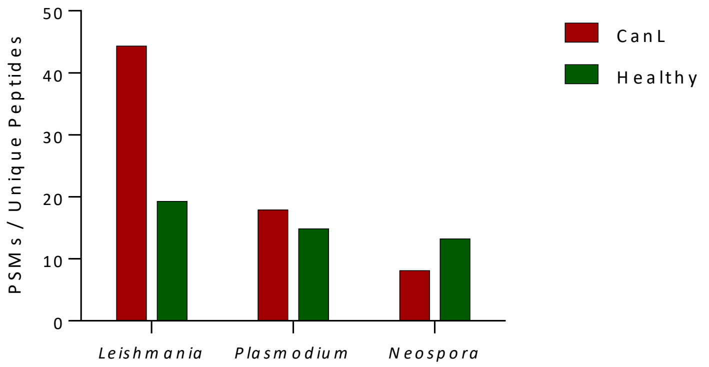

Figure S5. Number of PSMs per unique peptides identified for the two groups, Healthy and CanL, using different pathogens databases: *L. infantum, P. falciparum* and *N. caninum*.

Table S7. List of the *L. infantum* proteins found in more than one CanL sample. TriTrypDB accession number, Uniprot accession number, description and associated PSMs, unique peptides and peptide identification confidence, and corresponding number of dogs in which the protein was identified.

TriTrypDB Acession number	Uniprot Accession number	Description	PSMs	Unique Peptides	Confidence	Total number of dogs
LINF_140020900	A4HW91	Uncharacterized protein	1	1	Medium	4
			1	1	Medium	
			1	1	High	
			1	1	Medium	
LINF_160020100	A4HX38	Uncharacterized protein	1	1	Medium	3
			1	1	High	
			3	1	High	
LINF_190010100	A4HY74	Uncharacterized protein	6	1	High	2
			2	1	High	
LINF_280028700	A4I3U9	Uncharacterized protein	1	1	Medium	2
			1	1	Medium	
LINF_320042300	A4I8H2	Uncharacterized protein	1	1	Medium	2
			1	1	Medium	
LINF_330029800	A4I980	Putative copper-transporting ATPase-like protein	1	1	Medium	3
			3	1	Medium	
			2	1	Medium	
LINF_340007500	A4I9J4	Uncharacterized protein	2	1	Medium	2
			1	1	Medium	
LINF_340019300	A4I9T5	Uncharacterized protein	1	1	High	2
			2	1	Medium	
LINF_350022800	A4IB79	Putative DNA polymerase epsilon subunit b	4	1	High	6
			1	1	Medium	
			6	1	Medium	
			1	1	Low	
			2	1	Medium	
			1	1	Medium	
LINF_350034700	A4IBI2	Uncharacterized protein	3	1	Medium	2
			2	1	Medium	
LINF_360045700	A4ID28	Mitogen activated kinase-like protein	2	1	High	2
			1	1	Medium	
LINF_120011000	E9AGE5	Uncharacterized protein	8	1	High	2
			4	1	High	

Table S8. List of *L. infantum* proteins identified in more than one CanL dog, and respective UniProt accession number, peptide sequence identified and peptide blast result performed in NCBI.

Accession	Description	Peptides	Blast (100% match with *Leishmania* spp. / Match with other species)
A4HW91	Uncharacterized protein	[K].EESSSEEEELLVIGAVPLEFER.[L]	100% match with *Leishmania* spp.
A4HX38	Uncharacterized protein	[K].TLVSLQPK.[L]	100% match with *Leishmania* spp.
A4HY74	Uncharacterized protein	[R].YTADRVENTKNIIFWQHCR.[Y]	100% match with *Leishmania* spp.
A4I3U9	Uncharacterized protein	[R].TIALAQR.[E]	Match with other species
A4I8H2	Uncharacterized protein	[R].LPAAESAVR.[L]	Match with other species
A4I980	Putative copper-transporting ATPase-like protein	[R].IVQEAQNTKPSIQR.[A]	100% match with *Leishmania* spp.
A4I9J4	Uncharacterized protein	[R].EQVVVQLQREK.[G]	100% match with *Leishmania* spp.
A4I9T5	Uncharacterized protein	[R].SLIQLEADSK.[A]	100% match with *Leishmania* spp.
A4IB79	Putative DNA polymerase epsilon subunit b	[R].MQGLSESELR.[D]	100% match with *Leishmania* spp.
A4IBI2	Uncharacterized protein	[K].DILAVTR.[I]	100% match with *Leishmania* spp.
A4ID28	Mitogen activated kinase-like protein	[K].RGDALLK.[K]	Match with other species
E9AGE5	Uncharacterized protein	[K].KKMGYLGYELTAEEAAALR.[A]	100% match with *Leishmania* spp.

Figure S1. Clinical signs of the CanL dogs. Clinical signs are divided into unspecific, more general clinical signs (lymphadenopathy, epistaxis, weight loss and cachexia) and more specific clinical signs compatible with CanL—dermatological lesions, ophthalmological lesions, muscular skeleton lesions and haematological abnormalities. NA—not available. Figure S2. Reactivity of sera from CanL confirmed cases and healthy dogs for (a) SPLA and (b) rK39 determined by ELISA. Coatings were done with 10 μg/mL for SPLA and 5 μg/mL rK39. Results are represented as the logarithm of the optical density at 492 nm normalized by the cut-off value for each antigen. The dashed lines represent the cut-off. Data was generated from the average of two independent assays.

Figure S3. (a)—Results of BCA (mg/mL), bead-based assay for FACS analysis with CD5L, CD71 and CD9 and respective controls (mfi) for fractions 3–8 obtained from SEC. Control—beads + Fractions 5 and 6 + secondary antibody.

Figure S4. Protein content–EV characterization based on the Mysev2018. Each line represents a protein, while the columns labelled with a number represent the individual samples. The presence of green (Healthy group) or red colour (CanL group) associated with individual proteins depicted in lines represents the detection of that protein in the sample with at least 2 unique peptides. Figure S5. Number of PSMs per unique peptides identified for the two groups, Healthy and CanL, using different pathogens databases: *L. infantum, P. falciparum* and *N. caninum*. Table S1. Canine proteins identified in each sample. Each sheet in the Excel document corresponds to a different sample (samples 1 to 19—healthy samples; 20 to 39—CanL samples). Each protein is associated with the Protein FDR Confidence, Uniprot accession number, peptides, PSMs and unique peptides. Table S2. Canine proteins that were identified in each group. Each sheet in the Excel document corresponds to a different group: Healthy, CanL, core proteome, Unique to Healthy and Unique to CanL. The different proteins are associated with the Uniprot accession number, peptides, PSMs and unique peptides. Table S3. Gene ontology (GO) enrichment analysis of the proteins present in different groups. Each sheet in the Excel document corresponds to a different group: Healthy, CanL, core proteome, Unique to Healthy and Unique to CanL. GO enrichment analysis shows terms significantly

enriched for biological processes, molecular function and cellular compartment, corresponding genes to each GO term and respective *p*-value. Table S4. List of proteins identified in the injection analysis. Confidence associated with the peptide identification, Uniprot accession number and respective peptides, PSMs and unique peptides identified in each protein. Abundance ratio (CanL/Healthy) e respective *p*-value. The presence of green (Healthy) or red (CanL group) colours associated with individual proteins represents the detection of that PSM in the injection (corresponding to a technical replicate). Number of injections associated with Healthy group, CanL group, total number of injections and difference between the two groups. Table S5. List of accession numbers, description, fold change and *p*-value associated with the proteins significantly altered abundance in CanL EVs and respective peptides, Peptide-Spectrum Matches (PSMs) and unique peptides identified in Healthy and CanL EVs. Table S6. List of *L. infantum* proteins identified in CanL dogs. Respective TriTrypDB and Uniprot accession number, description, peptide sequence identified and peptide blast result performed in NCBI as well as peptides, PSMs, unique peptides and the total number of dogs associated with each protein. Table S7. List of the *L. infantum* proteins found in more than one CanL sample. TriTrypDB accession number, Uniprot accession number, description and associated PSMs, unique peptides and peptide identification confidence, and corresponding the number of dogs in which the protein was I dentified. Table S8. List of *L. infantum* proteins identified in more than one CanL dog, and respective UniProt accession number, peptide sequence identified and peptide blast result performed in NCBI
